# Editorial: Exploring immune memory dynamics in chronic antigen exposure and disease progression: implications for immunotherapy

**DOI:** 10.3389/fimmu.2026.1769061

**Published:** 2026-02-09

**Authors:** Stefano Caserta, Alejandra Pera

**Affiliations:** 1 Immune Cell Biology and Cancer Immunology Lab, Department of Cellular, Computational and Integrative Biology (CIBIO), University of Trento, Trento, Italy; 2 Centre for Biomedicine, Hull York Medical School, Faculty of Health Sciences, University of Hull, Hull, United Kingdom; 3 Maimonides Institute for Biomedical Research of Cordoba (IMIBIC), Córdoba, Spain; 4 Department of Cell Biology, Physiology and Immunology, University of Córdoba, Córdoba, Spain

**Keywords:** asthma, cancer, chronic antigen, CMV infection, immunological memory, sepsis, T memory stem cells, tissue resident memory cells

Immunological memory is a cornerstone of adaptive immunity, crucial for the long-term protection against pathogens. Traditionally, this has been attributed to the clonal expansion of antigen-(Ag)-specific T and B lymphocytes that provide rapid and effective responses upon pathogen re-exposure, constituting a foundational principle behind vaccination strategies (1).

Additionally, over the past decade, immune memory has emerged as a feature of innate immune cells, revealing striking complexity. Macrophages, monocytes, dendritic cells, and NK cells can undergo trained potentiation, enhancing responses to repeat infections and providing heterologous protection across different pathogenic insults. Furthermore, endotoxin tolerance (ET) originally characterised in 1947 (2) as decreased responsiveness to repeat stimuli is now considered a form of innate training by researchers proposing the term “trained tolerance” (3).

In this second issue of our collection (find here the first issue (4)), the enticing review of Lopez-Collazo and del Fresno compares trained innate immunity (TI) and ET. The triggers of TI (β-glucan; vaccines, *e.g. Bacillus* Calmette Guerin, influenza, and *Vaccinia*; adjuvants; flagellin and others) and ET (lipopolysaccharide, LPS; lipoteichoic acid; Pellino-3; erythropoietin; and mitochondrial DNA) are covered first, before delving into molecular mechanisms. A pivotal promoter of inflammation in hypoxia (5), HIF1α also drives ET, particularly under chronic more than acute stimulation, leading to activation of key negative regulators of inflammation (IRAK-M and PD-L1). The authors explore the impact of epigenetic reprogramming (possibly in linkage with metabolic changes) at the root of ET and TI. Ultimately, the identification of shared pathways would support the hypothesis that ET and TI represent two distinct states of innate memory, whether suppressive or not, and the interconversion between these would help future immunomodulatory pharmacotherapy approaches. Finally, the authors cover the fascinating concept of innate central memory, in which hematopoietic stem and progenitor cells of the bone marrow (BM) are trained by TLR or Dectin-1 ligands to imprint both states of innate immune memory in their myeloid progeny. Duration of innate immune memory differs between *in vitro* and *in vivo* approaches and lasts longer for TI over ET, including across generations [at least in mice (6, 7)]. How infectious cues are transmitted to the BM (and even gametes) remains unclear. The authors consider a series of implications of TI and ET for vaccine design, immunopathology and protection in stroke, COVID-19, sepsis and cancer.

TI does not only concern myeloid origin and NK cells: Verma et al. explore the molecular mechanisms behind memory ILC2 formation in a mouse model of asthma triggered by the fungal pathogen, *Alternaria alternata* (8). Memory ILC2 are developed after intranasal sensitisation with the fungal allergen extract. Severe allergy arises after recall responses, characterised by high bronchoalveolar infiltrates, rich in ILC2 effectors secreting IL-5 and IL-13, associated with lung tissue damage and NF-κB expression. Memory ILC2 express sets of repressor (*e.g.*, Zeb1, Nr4a2, Bach2, JunD, Fra1 and Fra2) and preparedness (e.g., Fhl2, Mpp7, Stat6, Srebf2) genes, with the latter typically inhibiting the expression of the repressors to favour memory development. The authors postulated that NF-κB would be ideally positioned to act as a candidate molecular switch between ILC2 repression/activation programs, given the dual nature of its structure. In fact, NF-κB contains two subunits (p50 and p65) that work in homodimers or heterodimers to elicit repression or activation of gene expression, respectively. Interestingly, the recall response of memory ILC2 effectors largely depends on NF-κB expression, given that genetic ablation of this transcription factor in knock-out mice lessens allergy, damage, and effector cytokine responses. Notably, training of memory ILC2 by sensitisation in the absence of NF-κB reduces effector cytokine potential before recall. This associates with the highest levels of expression of repressors, Zeb1, Nr4a2, Bach2, JunD, and, to a lower extent, preparedness genes (Fhl2 and Stat6). Nonetheless, in the presence of NF-κB, both repressor and preparedness genes are downregulated, more so after recall, possibly via the formation of NF-κB/RUNX1 heterodimers. This suggests that NF-κB mostly operates during the effector memory phase of ILC2 immunopathological responses in asthma, with attritional effects on memory cell formation.

Thus, persistent and/or recurrent Ag/pathogens can re-programme immune memory sustaining pathology rather than protection. Regarding this, Rodger et al. explore the role in health and disease of circulating T cells with tissue-resident memory phenotype (ex-TRM) preserving key residency markers (*e.g.*, CD103). Murine and human studies show that ex-TRM cells preferably home to their original tissue, displaying remarkable plasticity, differentiating into diverse memory/effector subsets. Ex-TRM contribute to homeostasis distributing pathogen-experienced cells across barrier sites. However, ex-TRM have also the ability to disseminate pathogenic tissue-primed responses acting as vectors of inflammation both locally and at distant sites. Thus, ex-TRM represent a critical intersection between local tissue-imprinted memory and systemic immunity, demonstrating how persistent/recurrent Ag stimulation at barrier sites can generate long-lived memory populations, capable of recirculating and spreading inflammation. The review finally highlights unknowns regarding the triggers, frequency, and regulation of TRM egress, and emphasises their potential as therapeutic targets.

Fazeli et al. thorough revision examines stem-cell memory T cells (TSCM) phenotype, differentiation pathways, metabolic features, and immunological functions, highlighting their unique stem-like properties and persistence. TSCM maintain autoreactive T-cell pools thanks to self-renewal and multipotent differentiation, showing longevity, resistance to exhaustion, and robust cytokine production. Evidence from multiple autoimmune diseases shows consistently increased frequencies of TSCM subsets that correlate with disease activity, treatment response, and persistence of pathogenic memory cells despite immunosuppression. The authors also discuss molecular and metabolic pathways (*e.g.*, Wnt–β-catenin, IL-7/IL-21 signalling, mitochondrial reprogramming) that support TSCM development and survival. Thus, TSCM cells pivotally sustain autoreactive responses over long periods, contributing to chronicity, relapse, and resistance to immunotherapy. The authors conclude that targeting TSCM cells could represent a promising therapeutic strategy and/or provide biomarkers across autoimmune conditions, while noting uncertainties regarding the consequences of reducing this subset.

Chronic cytomegalovirus (CMV) infection associates with T-cell memory inflation (9) linked with cardiovascular disease risk (10). In this context, Fuhrmann et al. aimed at determining if CMV broadly affects T-cell differentiation beyond CMV specificity. Comparison of older CMV-seronegative (CMV-) and CMV-seropositive (CMV+) individuals showed that only 20-30% of the CMV+ exhibit a marked expansion of terminally differentiated memory T cells. The degree of non-specific T cells differentiation weakly correlates with that of CMV-specific counterparts, indicating independent regulatory mechanisms. Logistic regression revealed that CMV serostatus exerts the strongest influence on memory subset composition, with smaller effects of age and sex. Thus, CMV-driven skewing of T-cell memory depends on unidentified predisposing factors that make certain individuals more susceptible to immune remodelling. This analysis highlights that chronic exposure does not uniformly drive exhaustion or terminal differentiation, underscoring the importance of individual susceptibility factors. Understanding memory cell differentiation under chronic stimulation is essential for predicting immune resilience, ageing trajectories, and responsiveness to immunotherapy.

Still on the theme of infection, sepsis is characterized by the establishment of an immunosuppressive state (known as immunoparalysis), driving the risk of secondary infections linked to late patient mortality (11). However, during the active infection stage of sepsis, immunosuppression may help restrain excessive inflammatory cytokine responses. In this respect, Anyalebechi et al. show that treatment of immunologically experienced septic mice with an agonist antibody (Ab) triggering the costimulatory molecule CD28 boosts sepsis survival through the activation of suppressive CD8+ T cells. CD28-agonist Ab promotes the generation of Foxp3+ CD8+ T cells that express high levels of suppressive IL-10 and inhibitory CTLA4. Upon CD8+ T cell depletion, mice survive less and suffer from augmented systemic cytokine release (high serum IL-1β, TNF, and IFNɣ) reminiscent of the cytokine storm. Interestingly, the same authors had previously shown that CD28-agonist treatment in naïve septic mice would instead dampen survival (12). Thus, this highlights the importance of previous Ag experiencing (or pre-existing immunity) in shaping the responses to sepsis, potentially key to survival, immunotherapeutic applications, and differences between childhood and adult sepsis. Suppressive CD8+ T cells were previously found to be generated upon exposure to superantigens (SAg) derived from bacteria frequently reported in sepsis (13), but it is still unclear whether these cells are protective. Evidence from Anyalebechi et al. suggests that Foxp3+ CD8+ T cells may be protective in sepsis, at least in mice.

Cancer immunosuppression and even certain anti-cancer treatments are associated with infections and sepsis. For instance, many patients live with chronic lymphocytic leukaemia (CLL) for decades, but they often suffer from repeat infections, while ultimately ~20% die of sepsis alone (14). BTK inhibitors (*e.g.*, ibrutinib) (15) that selectively target cancer cells still associate with infection occurrence. In this context, Tantoush et al. characterise the responses to staphylococcal SAgs to test whether exposure to the endemic *Staphylococcus aureus* (SA) compromises immunity in CLL patients. In an interesting link with Anyalebechi et al., Tantoush et al. show defective induction of Foxp3+ CD8+ and CD4+ T cells after exposure to SA SAgs (which notably stimulate the CD28 receptor) in CLL patients compared to healthy subjects. This may help explain the sustained cytokine responses detected in CLL patients after restimulation with SAgs. Thus, higher risk of developing sepsis in CLL individuals may derive from the heightened inflammatory milieu, exacerbated under the strain of specific microbial components that CLL patients may encounter. Tantoush et al. show that pseudo-exhaustion [*i.e.*: the maintenance of inflammatory cytokine responses in T cells that have acquired expression of inhibitory checkpoint receptors (16)] is enhanced upon staphylococcal SAg exposure, even more significantly in patients receiving ibrutinib treatment. Hence, pseudo-exhaustion typically described in CLL patients may result from lack of suppressive signals required to limit inflammatory loops activated by endemic pathogens, including SA. As to the tumour B cells, SA SAgs trigger activation of Ag-presentation and inflammatory potential of CLL cells, which adds to the inflammatory milieu. Hence, beyond increasing the risk of sepsis, the inflammatory activation of tumour cells by SA SAgs may drive cancer progression.

Still exploring the tumour microenvironment, Hou et al. characterise γδ T-cell memory subsets in younger adults (≤ 65 years) with non-M3 acute myeloid leukaemia (AML), evaluating associations with immune dysfunction and treatment success. Newly diagnosed AML patients showed a marked shift from central memory (CM) γδ T cells towards terminally differentiated effector memory T cells (TEMRA), alongside impaired function (reduced CD107a, IFN-γ, and perforin). AML patients displayed higher TIGIT expression across γδ T-cells, particularly within TCM cells, and partially normalised in complete remission. Higher frequencies of TIGIT+ γδ T cells and reduced TCM proportions were detected in BM compared to peripheral blood, highlighting the suppressive influence of the tumour microenvironment. Finally, authors identify TIGIT+ TCM γδ T cells as an independent predictor of poor responses to induction chemotherapy, proposing this subset as a potential target for immunotherapy. The profound skewing of TCR-γδ memory cell differentiation accompanied by exhaustion likely arise from persistent exposure to leukemic Ags that impair immunosurveillance. This study provides mechanistic and translational insights relevant for future immunotherapies targeting TIGIT in diseases shaped by chronic Ag exposure.

**Figure 1:**
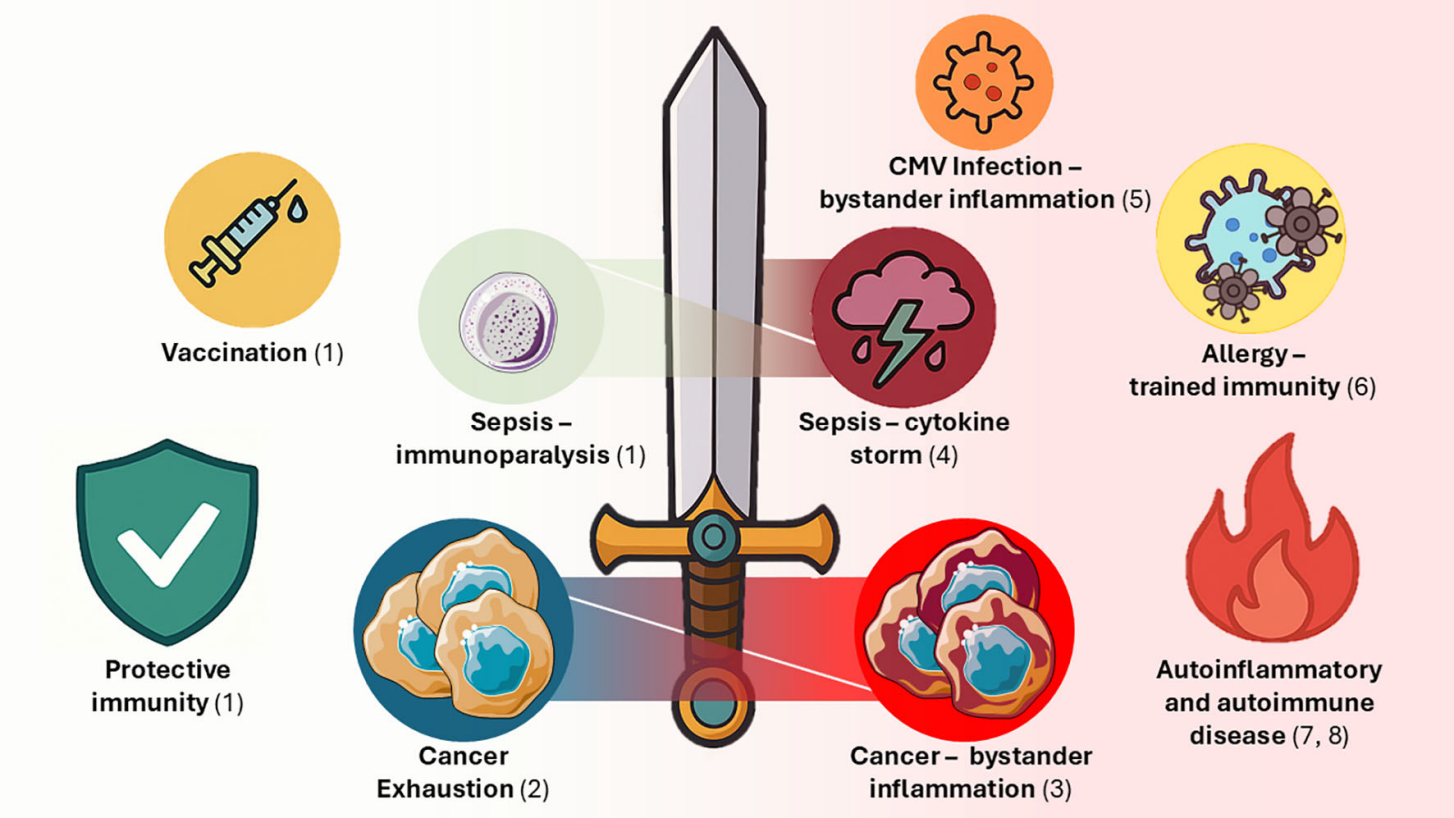
Immunological memory: a double-edged sword between protection and immunopathology. Immunity would benefit from long-term immunological memory, innate and/or adaptive, to protect from infections and disease (green shadowed area to the left side of the diagram). Lopez-Collazo and del Fresno reviewed the relationship between innate trained memory and endotoxin tolerance covering several sources that investigated the signals behind the fate of innate immune cells. Potentiation of trained immune memory (along with adaptive immunological memory) is amenable as part of vaccination strategies, in conditions associated with immunoparalysis (namely, in sepsis; see (1)) and/or exhaustion (as seen in cancer immunosuppression), in which long-term memory cells with lower levels of checkpoint inhibitory receptors are associated with better outcomes (covered by Hou et al.; see (2)). However, accumulation of immunological (innate and adaptive) memory cells is increasingly associated with immunopathological states (red shadowed area to the right side of the diagram). In Tantoush et al., pseudo-exhausted memory-like cells activate/accumulate in response to endemic pathogens supporting bystander inflammation in CLL, a frequent type of leukaemia; see (3). This favours an inflammatory tumour microenvironment which leads to the activation of CLL tumour cells, potentially promoting cancer progression. Likewise, in sepsis excessive inflammation supports cytokine storm responses, hence regulatory T cell that blunt the immune response are beneficial, especially in specific immunotherapy, as covered by Anyalebechi et al.; see (4). While accumulation of adaptive memory cells during CMV infection has been associated with increased CVD risk, the trajectories of memory cell accumulation vary from subject to subject as shown by Fuhrmann et al., with predisposing factors yet to be fully determined; see (5). Excessive innate/adaptive memory responses can ultimately drive severe pathology, including allergy, as seen as in the study by Verma et al. of memory ILC2 in asthma (see (6)), and autoinflammatory and autoimmune conditions, that can be driven by ex-TRM and TSCM cells, respectively reviewed in depth by Rodger et al. and Fazeli et al.; see (7) and (8).

In conclusion, this topic delved into the cellular and molecular mechanisms underlying immune memory responses to persistent/recurrent Ags, covering innate and adaptive immunity examples. While significant gaps remain in understanding how immunological memory is altered under specific conditions, such as autoimmune diseases, allergies, different types of cancer and infections, collectively the above studies provide meaningful insights. Ag/pathogen persistence can drive to memory cell exhaustion or trained tolerance, and reversal of these conditions is amenable in cancer, vaccination and even for sepsis immunoparalysis. However, risks arise from resetting the system with a bias towards excessive memory cell function, potentially leading to related immunopathology (**Figure 1**). At the opposite end of the spectrum, autoimmune disease, allergy and specific examples of infections (e.g., CMV) would benefit from switching off immune memory cells. In this respect, the identification of molecular switches and epigenetic modulators is key to open the way to pharmacological or immunotherapeutic interventions that could reverse memory states into suppression and *vice versa*. Still, there is a need to integrate the participation of innate and adaptive networks to obtain a thorough picture of the factors at play in immune memory balances, which would help identify the critical nodes that can be manipulated in future interventions.
